# Molecular and clinical profiles of syndecan-1 in solid and hematological cancer for prognosis and precision medicine

**DOI:** 10.18632/oncotarget.4981

**Published:** 2015-07-22

**Authors:** Mohamed R. Akl, Poonam Nagpal, Nehad M. Ayoub, Sathyen A. Prabhu, Matthew Gliksman, Betty Tai, Ahmet Hatipoglu, Andre Goy, K. Stephen Suh

**Affiliations:** ^1^ Genomics and Biomarkers Program, The John Theurer Cancer Center, Hackensack University Medical Center, Hackensack, NJ, USA; ^2^ Department of Clinical Pharmacy, Faculty of Pharmacy, Jordan University of Science and Technology, Irbid, Jordan; ^3^ Lymphoma Division, The John Theurer Cancer Center, Hackensack University Medical Center, Hackensack, NJ, USA

**Keywords:** syndecan, biomarker, cancer, personalized medicine, CD138

## Abstract

Syndecan-1 (SDC1, CD138) is a key cell surface adhesion molecule essential for maintaining cell morphology and interaction with the surrounding microenvironment. Deregulation of SDC1 contributes to cancer progression by promoting cell proliferation, metastasis, invasion and angiogenesis, and is associated with relapse through chemoresistance. SDC1 expression level is also associated with responses to chemotherapy and with prognosis in multiple solid and hematological cancers, including multiple myeloma and Hodgkin lymphoma. At the tissue level, the expression levels of SDC1 and the released extracellular domain of SDC1 correlate with tumor malignancy, phenotype, and metastatic potential for both solid and hematological tumors in a tissue-specific manner. The SDC1 expression profile varies among cancer types, but the differential expression signatures between normal and cancer cells in epithelial and stromal compartments are directly associated with aggressiveness of tumors and patient's clinical outcome and survival. Therefore, relevant biomarkers of SDC signaling may be useful for selecting patients that would most likely respond to a particular therapy at the time of diagnosis or perhaps for predicting relapse. In addition, the reciprocal expression signature of SDC between tumor epithelial and stromal compartments may have synergistic value for patient selection and the prediction of clinical outcome.

## INTRODUCTION

### Syndecan structure and expression

**Figure 1:**
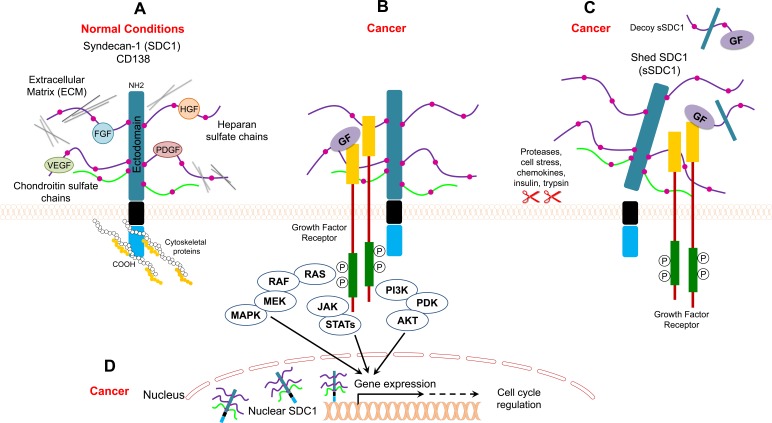
Model for SDC1 function under normal and cancer conditions **A.** SDC1 binds to ECM proteins and/or growth factors through its heparan sulfate chains, and it binds to cytoskeletal proteins for cell anchorage **B.** SDC1 acts as a coreceptor that facilitates interaction between growth factors and their receptors and enhances cancer mitogenic signaling **C.** Shed SDC1 (sSDC1) enhances the interaction between growth factors and their receptors in cancer or acts as a decoy receptor **D.** Nuclear SDC1 controls gene expression in cancer.

Syndecans are members of the transmembrane heparan sulfate proteoglycan (HSPG) family [1]. Mammals have four syndecan family members, designated as syndecan-1 (syndecan, SDC1, CD138) [2], syndecan-2 (fibroglycan, SDC2) [3], syndecan-3 (N-syndecan, SDC3) [4], and syndecan-4 (amphiglycan or ryudocan, SDC4) [5]. SDC1 is the most studied and best characterized member of the syndecan family. The protein structure of syndecan consists of extracellular, transmembrane, and cytoplasmic domains (Figure 1A). The large extracellular domain of syndecan is located on the N-terminus (ectodomain) and is comprised of glycosaminoglycan (GAG) chains (heparan sulfate and chondroitin sulfate) [6]. All syndecans are anchored to the plasma membrane via a 24-25 amino acid hydrophobic transmembrane domain, which is highly conserved among the four syndecans. The cytoplasmic domain of syndecan contains the C-terminus, which is relatively short and comprised of 28-34 amino acids (Figure 1A). Importantly, the cytoplasmic domain of syndecan can be linked to intracellular cytoskeletal elements that maintain cell shape and provide support to the cytoskeleton (Figure 1A) [6–8]. In mammalian cells, the expression of syndecans is tightly regulated and, in turn, may control many downstream signaling events. Syndecans show different patterns of expression in various tissues. While SDC1 is predominantly expressed in epithelial and mesenchymal cells, SDC2 is the most abundantly expressed in cells of mesenchymal origin than in neuronal and epithelial cells. In hematopoietic tissues, SDC1 is predominantly expressed on the cell surface of immature B cells and mature plasma cells [9]. SDC3 is mainly expressed in neuronal and musculoskeletal tissue, whereas SDC4 is ubiquitously expressed [10]. Evidence indicates that the expression of SDC1 can be regulated by multiple growth factors, such as tumor growth factor-β (TGF-β) and basic fibroblast growth factor (bFGF or FGF2), in different mammalian cell types [11]. Tumor necrosis factor-α (TNF-α) downregulates SDC1 expression in endothelial cells [12], whereas TGF-β2 downregulates SDC1 expression in epithelial cells [13]. In addition, SDC1 expression is highly increased during the wound repair process [14].

### Syndecan localization and function

#### Cell surface syndecan

Syndecan (originating from the Greek word *syndein,* meaning “to bind together”) acts as an anchor to stabilize the morphology of epithelial sheets by connecting the extracellular matrix to the intracellular cytoskeleton [15]. Syndecan is expressed on the surface of all adherent cells and on many non-adherent cells [15]. It is well-established that syndecan serves as coreceptor for various heparin-binding growth factors, such as bFGF/FGF2, vascular endothelial growth factor (VEGF), TGF-β, and platelet-derived growth factor (PDGF) (Figure 1A, 1B) [16–18]. The interaction between syndecan and growth factors is facilitated through heparan sulfate (HS) chains (Figure 1A). In this regard, HS chains serve as templates that bridge growth factors and their receptors. In the case of FGF, syndecan acts as coreceptor to enhance the binding between FGF and the FGF receptor. Such binding lowers the concentration of FGF required to initiate downstream signaling through its receptor and extends the duration of receptor signaling (Figure 1B) [19]. In addition to its role as a coreceptor, syndecan itself acts as receptor via its HS chains (Figure 1A). Syndecan binds to different matrix elements through interactions with heparan-binding molecules on adjacent cells to potentiate cell-matrix adhesion (Figure 1A) [20–22]. Examples of extracellular molecules that commonly bind to syndecan in order to mediate cell adhesion to the extracellular matrix include collagens, fibronectin, thrombospondin, and tenascin [20–22].

Unlike HS chains, the biological function of chondroitin sulfate (CS) chains present in syndecan is not completely understood. A study by Okamoto and colleagues suggested a cooperative role of CS chains with HS chains in binding to the extracellular matrix protein laminin [23]. Although HS chains are major contributors to the function of syndecan, recent studies have revealed that the protein core ectodomains are also engaged in protein-protein interactions between syndecan and other peptide molecules [24, 25]. On the intracellular side, the cytoplasmic domain binds to several cytoskeletal proteins, such as ezrin, tubulin, and cortactin, which potentiates cell anchorage and stabilizes cell morphology [6–8]. Thus, syndecan plays an important role in the interplay between target cells and the extracellular matrix. Among the different syndecans, SDC1 was first to be identified and evaluated and is implicated in the maintenance of epithelial morphology and anchorage-dependent growth [26, 27].

#### Shed/Soluble syndecan

Syndecan can be proteolytically cleaved at a juxtamembrane site, which releases the extracellular (ectodomain) core protein bearing both HS and CS chains (Figure 1C) [28]. Cells constitutively shed syndecan at low levels, but shedding is accelerated in response to growth factors, chemokines, heparanase, microbial toxins, insulin, and cellular stress [29, 30]. These stimuli trigger several signaling pathways that eventually lead to elevated protease activity driving syndecan shedding. The syndecan molecules that are shed remain biologically active and can bind the same ligands as the intact ectodomain. Accordingly, shed syndecan may act in a paracrine manner [31]. On the other hand, shed ectodomains may compete for the same ligand as the surface receptor (acting as decoy receptors), thus downregulating signal transduction (Figure 1C) [31].

### Syndecan in Cancer

The expression of SDC1 is dysregulated in cancer, and low expression of SDC1 in epithelial cells is associated with poor prognosis and high metastatic potential [32–34]. Tables 1 and 2 summarize the studies to date that have evaluated SDC1 prognostic significance and clinical implications in solid and hematological tumors, respectively.

**Table 1:** Studies evaluating SDC1 as a prognostic biomarker in cancer patients with solid tumors

Cancer Type	Patient Number	Specimen Type	Cancer Subtype	Location (Method)	SDC1 Expression Pattern	Prognosis	Associated with	Ref
**Bladder**	109	Surgical resection	NMIBC	Epithelial (IHC)	(↓) in tumor vs. normal urothelium	(↓) RFS in low vs. high expression (p<.001)	High-stage disease	[64]
	198 vs.15 controls	Untreated surgical	Urothelial	Epithelial (IHC)	(↓) with tumor stage (p<0.001) and tumor grade (p< 0.001)	(↓) DSS in negative vs. positive expression (p=.021)	Epithelial with low stage, low-grade; stromal reciprocally associated; serum with high-stage, high-grade, metastases	[65]
				Stromal (IHC)	(↓) with tumor stage (p=0.001) and tumor grade (p=0.004)	(↓) DSS in positive vs. negative expression (p<0.001)		
				Serum (ELISA)	(↓) in metastatic stage (p<0.001)	(↓) DSS in high (≥91 ng/mL) vs. low (<91 ng/m) levels (p=0.075)		
	*Urine:* 102 vs. 206 controls; *tissue:* 185 vs. 8 controls	Voided urine and archived tumor blocks	NMIBC and MIBC	Epithelial (IHC)	(↓) in membranous and (**↓)** in cytoplasmic SDC1 in high grade tumors vs. low-grade tumors (p<0.0001)	(↓) DSS in cytoplasmic vs. membranous expression (p=0.0001)	Epithelial with high-grade, high stage tumors, poorly differentiated; urine with tumor grade, invasive-type	[66]
				Urine (ELISA)	No significant difference tumor vs. control			
**Breast**	80	Irradiated and hormone treated surgical	Invasive ductal	Epithelial (IHC)	61.25% tumors positive (cutoff: >10% positive cells)	(↓) ∼0.6 fold 7-yr RFS in epithelial-/stromal+ vs. epithelial+ and/or stromal expression (p<0.0001)	High epithelial expression is associated with low-histological grade, well-differentiated tumors	[70]
				Stromal (IHC)	(↓) in cancer (30%, cutoff: >10% of cells positive) vs. absent in control			
	62 vs. 10 controls	Excisional biopsy	Invasive ductal and lobular	Epithelial (IHC)	(↓) in tumor vs. control (breast carcinoma patients without metastasis, p<0.0001)	(↓) OS (p=0.02) and DSS (p=0.01) in high vs. low epithelial expression	Histological grade; inversely with hormonal receptor status	[72]
				Stromal (IHC)	83% tumor positive vs. 100% control	-		
	200	Surgical	Invasive ductal, lobular, medullary, papillary, mucinous	Epithelial (IHC)	61% tumors positive (cutoff: >5% positive cells)	(↓) 0.7 fold 10 yr.- OS in epithelial+/stromal+ vs. epithelial- and/or stromal expression (p<0.002)	DNA ploidy, ER-positive, cytoplasmic staining associated with poorly differentiated tumors	[76]
				Stromal (IHC)	67% tumors positive (cutoff: >5% positive cells)			
	127	Surgical	DCIS	Epithelial (IHC)	(↓) in low vs. high grade tumors (p=0.043)	No significant difference	ER, PR negative status	[71]
	207	Surgical	_	Epithelial (IHC)	(↓) in high grade (p= 0.0007)	(↓) OS in high SDC1 IHC score (>6.5) vs. low (<6.5) (p= 0.013)	Tumor size, grade, metastasis, ER status	[74]
	102	Surgical resection	Primary invasive	Epithelial (IHC)	73.5% tumors positive (>80% positive cells) vs. absent or weak in controls	(↓) OS in strong vs. weak SDC1 staining intensity (p=0.041, n=98)	Inversely with PR	[75]
				Stromal (IHC)	29.5% tumors positive	No significant difference		
**Cervical**	121	Surgical resection	_	Epithelial (IHC)	83.5% tumors positive	(↓) proportion surviving in negative, weak positive vs. strong staining (p=0.0219)	High epithelial expression is associated with low-histological grade	[77]
	244	Untreated surgical	Intraepithelial, microinvasive, invasive	Epithelial (IHC)	Loss of expression in tumors vs. basolateral in normal cervix	No significant difference	Squamous histology, metastatic lymph nodes	[78]
	124	Untreated surgical	SCC, ACA	Epithelial (IHC)	36% tumors strong positive	No significant difference	Differentiation grade, squamous histology	[79]
**Colorectal**	158 vs. 15 adenomas and 14 controls	Diagnostic biopsy	ACA	Epithelial (IHC)	(↓) (29% patients negative) or loss vs. normal epithelia	No significant difference	Poor differentiation, TNM staging, lymph node metastasis	[81]
				Stromal (IHC)	16.6% tumors positive vs. negative normal stroma			
**Endometrial**	109	Surgical	ACA, adenosquamous and serous carcinomas	Epithelial (IHC)	58% tumors strong positive and (↓) in advanced stage vs. early stage (p=0.007)	(↓) estimated 5-yr DFS and OS in low epithelial and high stromal (P<0.0001)	Advanced stage, high grade, lymph node metastasis	[83]
				Stromal (IHC)	16% tumors strong positive and (↓) in high grade tumors (p=0.05)			
**Gallbladder**	43	Untreated surgical resection	ACA	Epithelial	58.1% tumors positive vs. absent in controls	(↓) 0.5 fold 5-yr OS in positive vs. negative expression (p=0.05)	Lymph node metastasis, lymphovascular invasion	[85]
				Stromal	No stromal expression	-		
**Gastric**	337	Surgical	ACA	Epithelial (IHC)	31% tumors positive	(↓) 0.5 fold cumulative 5 yr survival in negative vs. positive expression (p=0.002)	High grade, nodal metastases	[86]
				stromal (IHC)	9% positive	(↓) 0.5 fold cumulative 5 yr survival in positive vs. negative expression (p=0.038)		
	296	Surgical	ACA	Epithelial (IHC)	47% tumors positive (cutoff: >60% cells), 26% negative	(↓) ∼0.5 fold cumulative 5-yr survival in low vs. high expression (p=0.0012)	Loss of epithelial SDC1 is correlated with high TNM stage, lymph node metastases, large tumor size	[87]
				Stromal (IHC)	9% positive	(↓) ∼0.5 fold cumulative 5-yr survival in high vs. low expression (p=0.0193)		
**Glioma**	116 vs. 15 controls	Surgical resection	**_**	Epithelial (qRT-PCR, IHC, Western blot)	(↓) >2 fold gene expression in tumors vs. controls; (↓) >3 fold protein expression in tumors vs. controls (p<0.001), 82.8% positive for Immunostaining (p<0.001)	(↓) OS in SDC1 positive vs. negative expression (p= 0.006)	Advanced tumor progression	[88]
**Head and Neck**	175	Post-operative irradiated surgical	Primary SCC	Epithelial (IHC)	52.6% tumors strong positive (>50% cells)	(↓) 0.7 fold 2-yr. OS in weak positive (cutoff: <50% cells positive) vs. strong positive (>50% cells positive), p=0.001)	Loss of epithelial SDC1 is associated with large tumor size, positive nodal status, high clinical stage	[110]
	29	Untreated diagnostic biopsy	Primary SCC	Epithelial (IHC)	(↓) SDC1 expression in poor differentiated tumors (25% patients) vs. well differentiated (p=.006), strong expression in normal oral mucosa	(↓) 0.3 fold 2-yr OS in weak or no expression vs. strong positive (p=0.001); (↓) 0.3 fold 2-yr RFS in weak expression vs. strong positive (p=.003)	Poorly differentiated tumors, large tumor size	[111]
**Laryngeal**	100	Tissue blocks	Glottic, supra glottic, and transglottic	Epithelial (IHC)	45% tumors strong positive	(↓) 0.8 fold 5-yr OS weak or no expression vs. strong expression (p=.048)	Poor differentiation	[90]
**Liver**	295	Serum	Barcelona clinic liver cancer	Soluble	(↓) in patients vs. low in patients without HCC or early HCC (p<0.0001)	(↓) OS in high (>50ng/ml) vs. low (<50ng/ml) levels (p=0.006), (↓) RFS in high vs. low levels (p=0.025)	Advanced stages of primary liver tumors	[91]
	57	Biopsy, surgical resection, autopsy	With intra or no intra hepatic metastatic lesion	Epithelial (IHC, Northern blot)	31.6% tumors positive, (↓) gene expression in tumors vs. normal epithelia	_	Poor differentiation, intra-and extra hepatic metastases	[92]
	37	Surgical resection	ACA	Epithelial (IHC)	(↓) in tumors vs. non-neoplastic liver, (↓) in poor differentiated tumors vs. well differentiated (p<0.01)	(↓) 5-yr survival in negative vs. positive (p<0.01), (↓) 0.6 fold 3-yr survival in negative vs. positive (p<0.01)	Poor histological differentiation, lymph node metastases	[34]
**Lung**	184 vs. 100 controls	Untreated diagnostic biopsy, serum	NSCLC and SCLC	Soluble (ELISA)	(↓) 2.5 fold in tumors vs. normal controls (p< 0.0001)	(↓) ∼2-fold months survival in higher vs. lower expression than median (median= 41ng/ml, p=0.0030)	Large tumor mass, advanced cancer	[93]
	116	Surgical resection	SCLC	Epithelial (IHC)	(↓) in poor differentiated tumors (22%, cutoff: <10% positive cells) vs. well differentiated (63%, p=.001)	(↓) 0.6 fold 5-yr OS in low vs. high expression (p=0.026)	Poorly differentiated	[94]
**Mesothelioma**	*Pleural effusions:* 163 vs. 93 benign; *sera*: 165 vs. 66 benign	Untreated pleural effusion and serum	_	Soluble (ELISA)	(↓) in malignant pleural effusion vs. control (p < 0.0001). No significant difference in sera	(↓) 0.5 fold survival in higher vs. lower than median SDC1 levels (median 100.2 ng/ml, p=0.005).	Metastases	[164]
	20, 57 (other cancers), 20 controls	Tissue blocks	Epithelial, biphasic, sarcomatoid mesothelioma	Epithelial (IHC)	(↓) in cancer vs. controls	(↓) survival in low (<25% immunoreactivity) vs. high expression (p=.033)	_	[95]
**Nasopharyngeal**	68	Untreated biopsy		Epithelial (IHC)	21% tumors positive (>50% cells) and 52% negative (<10% cells)	(↓) 0.6 fold 5-yr OS in positive vs. negative expression (p=0.04)	_	[96]
**Oral**	79	Archived tissues	Ameloblastomas, KCOT, dentigerous cysts	Epithelial	(↓) in ameloblastomas (26.3%) vs. KCOT (92.3%), dentigerous cysts (100%)	_	Lesions’ extension, involvement of adjacent structures	[98]
				Stromal (IHC)	No significant difference			
	72	Untreated biopsy	SCC	Epithelial	9.7% tumors showed strong staining, 50% weak or negative staining vs. strong staining in normal epithelium	(↓) 0.7 fold DFS in weak vs. strong or intermediate SDC1 intensity (p=.0138)	Nodal metastasis, differentiation, IFG score	[100]
	39	Untreated biopsy excision	SCC	Epithelial (IHC)	(↓) in tumors vs. controls (strong intensity in 15.4% patients, weak in 2%)	-	Recurrent events, rapid tumor progression	[101]
				Stromal (IHC)	(↓) in tumors vs. normal epithelium	(↓) 2-yr survival in stromal positive vs. negative expression		
**Ovarian**	111	Surgical	ACA	Epithelial (IHC)	(↓) in advanced stage tumors vs. early stage (p=0.01)	(↓) estimated 5-yr PFS (p=0.025) in negative vs. positive epithelial SDC1	Advanced stage	[103]
				Stromal (IHC)	(↓) in advanced stage vs. early stage tumors (p< 0.0001)	(↓) estimated 5-yr PFS (p=0.001); OS (p=0.022) in high vs. low stromal expression	Histological subtype, massive ascites, lymph node metastasis	
	115 vs. 10 borderline, 10 benign, 12 controls	Archived surgical	ACA	Stromal (IHC)	Positive expression in tumors vs. absent in normal ovaries	(↓) 10-yr PFS (p=0.005); OS (p=0.027) in strong vs. weak or no stromal expression	Advanced stage	[104]
**Pancreatic**	144 vs. 18 pancreatitis, 4 controls	Surgical	Ductal ACA	Epithelial (IHC)	94% tumors positive (cutoff: >20%)	(↓) 0.2 fold cumulative survival in stromal+/epithelial- vs. stromal-/epithelial+ and other patients (p=0.003)	Epithelial: better prognosis Stromal:histological grade, tumor location	[105]
				Stromal (IHC)	(↓) in tumors vs. acute and chronic pancreatitis samples			
**Prostate**	60 vs.10 controls	Surgical	Localized	Epithelial (IHC)	(↓) membranous and (↓) cytoplasmic (64% tumors) vs. benign prostatic hyperplasia, tonsil cancer samples	(↓) PSA-RFS in altered vs. normal SDC1 expression (p<0.05)	High Gleason score	[106]
	232	Untreated surgical	Localized	Epithelial (IHC)	(↓) cells expressing SDC1 (37.1% tumors positive) vs. normal adjacent and benign prostatic basal cells	(↓) PSA-PFS in positive vs. negative expression (p=0.034)	Higher PSA levels, lymph nodes metastases	[107]
	551 vs. 83 controls	Surgical	Localized	Epithelial (IHC)	(↓) in 36.7% tumors positive vs. 22.9% controls	(↓) time to progression (p<0.02), tumor specific survival (p<0.01), OS (p=0.07) in positive vs. negative expression	High Gleason grade	[109]
**Thyroid**	62	Surgical	PCT	Epithelial (IHC)	Strong in PCT-E vs. PCT-NE (p=.002)	_	Extracapsular invasion, tumor progression.	[165]
				stromal (IHC)	Strong in PCT-E vs. PCT-NE (p=.048)			

ACA: Adenocarcinoma; DCIS- Ductal carcinoma in-situ; DFS- disease free survival; DSS- disease specific survival; ER- estrogen receptor; HCC: hepatocellular carcinoma; IFG – Histological grade of malignancy at deep invasive front; IHC- immunohistochemistry; KCOT- keratocystic odontogenic tumor; MIBC- muscle invasive bladder cancer; NMIBC- Non-muscle invasive bladder cancer; NSCLC- Non-small cell lung cancer; OS- Overall survival; PCT-E - Papillary carcinomas of the thyroid with extracapsular extension; PCT-NE - Papillary carcinomas of the thyroid without extracapsular extension; PFS –Progression free survival; PR- progesterone receptor; RFS- Recurrence free survival; PSA- prostate-specific antigen; SCC- squamous cell carcinoma; SCLC- small cell lung cancer; SDC1: syndecan-1.

**Table 2:** Studies evaluating SDC1 as a prognostic biomarker in cancer patients with hematological tumors

Cancer Type	Patient Number	Specimen Type	Cancer Subtype	Location (Method)	SDC1 Expression Pattern	Prognosis	Associated with	Ref
**Chronic lymphocytic leukemia**	104 vs. 32 controls	Blood and BM samples	_	Soluble (ELISA)	(↓) in tumors vs. controls	(↓) 0.6 fold OS in high (>53 ng/ml) vs. low (<53 ng/ml) serum SDC1 level (p=0.0004)	No significant correlation	[112]
**Diffuse large B-cell lymphoma**	46	Untreated biopsy	Nodal, extra-nodal	Epithelial (IHC)	Positive in 39% tumors	(↓) OS in positive vs. negative SDC1 expression (p=0.01)	_	[114]
	51	TMA blocks	Nodal, tonsillar	Epithelial (IHC)	Positive in 15.7% tumors	(↓) OS in positive vs. negative SDC1 expression (p=0.0008)	_	[113]
**Hodgkin's lymphoma**	25	Blood, tissue blocks	NS	Epithelial (IHC)	(↓) in HL tumors vs. NHL and normal lymph nodes, 56-fold increase in poor vs. good outcome	Poor outcome	_	[138]
	66	Untreated serum	NS, MC, LP	Soluble (ELISA)	(↓) ∼1.5-fold in HL vs. NHL and lymph node controls (p<0.001)	No significant difference	Age, gender, elevated B2M and IPS	[124]
**Multiple myeloma**	17 MM, 14 MGUS	Serum	_	Soluble (ELISA)	(↓) ∼48-fold in MM tumors vs. MGUS (p<0.001)	(↓) OS in high (>500 ng/ml) vs. low (<500 ng/ml) level (p=0.029)	_	[119]
	478	Serum	_	Soluble (ELISA)	(↓) ∼1.7-fold in presentation phase vs. plateau phase patients	(↓) 0.7 and 0.5 fold years survival in high (>960 ng/ml) vs. low (<196 ng/ml) level; (p=0.0006)	Monoclonal paraprotein, B2M, BM plasma cell content	[56]
	67	Untreated blood, BM aspirates or biopsy	_	Soluble (ELISA)	(↓) ∼13-fold in tumors vs. controls (p <0.0001); (↓) 2 fold in BM vs. blood (p<0.0001)	(↓) 0.7 fold OS in high (>225ng/ml) vs. low (<225ng/ml) (p=0.039)	Microvessel density	[121]
	32 vs. 11 controls	Untreated, post remission serum	_	Soluble (ELISA)	(↓) in pretreatment serum (p=0.001) and (↓) in treated subjects vs. healthy controls	(↓) 0.5 fold 5-yr OS in high (>75ng/ml) vs. low (<75ng/ml) serum SDC1 levels (p=0.01)	Stage and B2M	[122]
	25 vs. 10 controls	Untreated and treated serum	_	Epithelial (FCM)	(↓) responders and survivors vs. nonresponders and nonsurvivors (P < 0.01 )	(↓) survival in low vs. high cellular expression (p<0.01)	Inversely: with stage; directly: with plasma cell count, B2M, serum creatinine, CRP, AP and serum calcium	[116]
				Soluble (ELISA)	(↓) ∼9-fold in tumors vs. controls (p <0.001), high in non-responders and non-survivors	(↓) survival in high vs. low serum SDC1 levels (p<0.001)		
	35 vs. 21 (other diseases), 14 controls	Untreated BM aspirates	_	Soluble (ELISA)	(↓) >2-fold in cancer vs. other diseases subjects (p>0.002 ) and healthy controls (p>0.001)	_	HGF	[117]

AP: alkaline phosphatase; Beta-2 microglobulin (B2M); BM: bone marrow; CRP: C-reactive protein; DLBCL: diffuse large B cell lymphoma; FCM: flow cytometry; HGF: hepatocyte growth factor; IHC: immunohistochemistry; IPS: International Prognostic score; LP: lymphocyte predominance; MGUS: monoclonal gammopathy of undetermined significance; MC: mixed cellularity; MM: multiple myeloma; NHL- Non-Hodgkin Lymphoma; NS: Nodular sclerosis; OS: overall survival; SDC1: syndecan-1; TMA: tissue microarray.

Studies have shown that SDC1 is involved in multiple cellular processes, including cell proliferation [24, 39–41], migration [42–44], adhesion, and angiogenesis [30, 45]. In general, the loss of SDC1 expression in carcinoma cells reduces cell adhesion to the extracellular matrix and enhances cell motility and invasion [15]. Alternatively, increased stromal SDC1 expression alters fibronectin production and extracellular matrix organization [35]. In addition, increased expression of SDC1 in stromal fibroblasts is associated with angiogenesis and cancer progression [15, 36]. Various signaling molecules function upstream or downstream from SDC1 in cancer (examples are listed in Table 3). SDC1 acts as a scaffold that brings ligands, such as hepatocyte growth factor (HGF), bFGF/FGF2, and VEGF, in close proximity to their cognate receptors. This localization activates downstream signal transduction pathways, such as the “PI3K to Akt” and “Ras to MAPK” pathways, which enhances the proliferation of endothelial cells, cancer cells, and fibroblasts (Figure 1B) [15, 37]. For example, the binding of HGF with SDC1 enhances downstream signaling in myeloma cells, osteoblasts, and stromal cells [15, 38]. SDC activates integrin αvβ [46] and Wnt5a [47] signaling in breast cancer and multiple myeloma, respectively (Table 3). SDC increases cell adhesion via activation of focal adhesion kinase (FAK) signaling in lung and colorectal cancers [48, 49]. Mulitple molecules such as ADAM-10, ADAM-17, MMP-7, MMP-9, MMP-14, and bFGF/FGF2 increase SDC1 shedding in multiple myeloma as well as breast and colon cancers [50–54] (Table 3).

**Table 3:** Examples of proteins associated with the SDC pathway in cancer

Protein	Association	Level of Interaction	Cancer	Pathway	Ref
ADAM10, ADAM17	ADAM10 and ADAM17 contribute to SDC1 shedding by HVECs in response to TSST-1	Upstream	Multiple myeloma	TSST-1	[50]
αvβ3	SDC1 increases αvβ3 integrin activation and signaling	Upstream	Breast cancer	avΔ3 integrin	[46]
	αvβ3 integrin forms a complex with extracellular domain of SDC1 which promotes docking of IGFR with SDC1 ectodomain	Upstream	Breast cancer	avΔ3 integrin	[166]
BCL-2	SHH signaling enhanced SDC+ cell proliferation and activated BCL-2, leading to the inhibition of cancer cell apoptosis	Upstream	Multiple myeloma	Hedgehog	[167]
bFGF	bFGF increases SDC1 shedding, and SDC1 induces bFGF signaling	Upstream	Pancreatic cancer	FGFR	[54, 133, 134]
CASK	SDC2 and 3 associate via their C-terminal binding motif with CASK	Downstream	Breast cancer	Cask signaling	[168]
FAK	Cancer cells contact with fibronectin leads to SDC2 upregulation and increase in cell adhesion via FAK signaling	Downstream	Colorectal cancer	FAK	[49]
Fibronectin	The trimeric Tn antigen on SDC1 enhances integrin α5β1 functions, resulting in increased adhesion to fibronectin via the FAK/paxillin pathway	Upstream	Lung cancer	FAK	[48]
IGF1R	IGF1R, avΔ3 integrin and SDC1 form a ternary receptor complex in which signaling downstream of IGF1R activates avΔ3 integrin	Upstream	Breast cancer	IGF1R	[139]
KRAS	SDC2 cooperates with KRAS to induce an invasive phenotype	Downstream	Pancreatic cancer	KRAS /MAPK	[169]
MMP-7	SDC2 elevates MMP-7 expression, and MMP-7 increases SDC2 shedding	Downstream	Colon cancer	Wnt	[51]
MMP-9	Increased expression of MMP-9 lead to increased shedding of SDC1	Upstream	Multiple myeloma	ERK Signaling	[52]
MT1-MMP (MMP-14)	MT1-MMP expressed by stromal fibroblasts cleaves SDC1 and releases SDC1 ectodomain as a paracrine mediator	Upstream	Breast cancer	Multiple	[53]
VEGFR	Shed SDC1 forms a complex with VEGF which activates VEGFR receptors	Upstream	Multiple myeloma	VEGFR	[30]
Wnt5a	SDC1 and SDC4 increase Wnt5A signaling and cellular invasion	Downstream	Multiple myeloma	Wnt	[47]

ADAM: A disintegrin and metalloproteinase domain-containing protein; αvβ3: alpha-v beta-3; BCL-2: B-cell lymphoma 2 protein; bFGF: basic fibroblast growth factor; CASK: calcium/calmodulin-dependent serine kinase; FAK: focal adhesion kinase; HVECs: human vaginal epithelial cells; IGF1R: insulin-like growth factor-1 receptor; MMP: matrix metallopeptidase; MT1-MMP: membrane type 1 matrix metalloproteinase; SDC: syndecan; SHH: hedgehog signaling pathway; TSST-1: toxic shock syndrome toxin-1; VEGFR: vascular endothelial growth factor receptor

Alterations in the levels of soluble SDC1 have been reported in various cancer types. The levels of soluble SDC1 in the sera of healthy persons are relatively low compared to levels in cancer patients. Heparanase induces SDC1 shedding, and soluble SDC1 is an independent negative prognostic factor in multiple myeloma [32, 55, 56]. High levels of heparanase have also been reported in the plasma of Hodgkin's lymphoma (HL) patients and it can be used to evaluate treatment response [57]. Soluble SDC1 is biologically active and can intensify the binding between growth factors with their receptors in tumor stroma [30, 58]. Soluble SDC1 ectodomains bind to pro-angiogenic factors, which promotes endothelial cell invasion (Figure 1C) [30]. Soluble SDC1 also increases fibroblast proliferation and the release of TGF-β [58]. In addition, soluble SDC1 can act as decoy receptor, and thus it may promote cancer progression by sequestering inhibitory molecules (Figure 1C) [59].

Many reports indicate that Heparan Sulfate Proteoglycans (HSPGs) may localize to the nucleus [60, 61]. A recent study showed that SDC1 is present in the nucleus of myeloma tumor cells where it activates gene transcription (Figure 1D) [62]. In addition, shed SDC1 can localize to the nucleus in areas involved in gene transcription [63].

### Syndecan as a prognostic biomarker in solid tumors

#### Bladder cancer

High SDC1 expression was observed in over 60% of specimens from patients diagnosed with primary non-muscle-invasive bladder cancer, and SDC1 was a significant predictor for recurrence-free survival [64]. In addition, SDC1 was found to be expressed on the cell membrane of normal bladder epithelium and non-muscle-invasive bladder cancer cells, but was almost completely absent in muscle-invasive carcinomas [65]. In contrast, stromal SDC1 as well as serum SDC1 levels were higher in muscle-invasive compared to non-muscle-invasive bladder cancer cells. Lymph node-positive cases had the highest SDC1 serum concentrations, and SDC1 expression in stromal cells was independently associated with survival. Loss of SDC1 in tumor cells and the simultaneous increase of serum SDC1 levels in high-stage, high-grade bladder cancer cells suggest that SDC1 shedding is associated with bladder cancer cell aggressiveness [65]. Thus, circulating levels of SDC1 may ultimately be a useful prognostic tool for identifying patients with lymph node metastases [65].

Assessing urinary SDC1 levels and tumor SDC1 expression revealed no significant difference in urinary SDC1 levels between cancer and healthy subjects [67]. However, urinary levels of SDC1 were reduced in high-grade disease compared to low-grade disease states [66]. Interestingly, SDC1 predominantly localized to the cell membrane in normal tissue and low-grade tumors, while high-grade tumors exhibited distinct cytoplasmic localization. In these reports, the tumor stage and grade can change the value of urinary and serum levels of SDC1 as prognostic tools in urinary bladder cancers. However, localization of SDC1 in the membranous or cytoplasmic compartments may correlate with the stage and aggressiveness of urinary bladder cancers.

#### Breast cancer

SDC1 has been shown to be expressed at high levels in breast cancer specimens and was associated with high histologic grade, large tumor size, high mitotic count, and poor prognosis [67]. High SDC1 levels were also associated with a higher risk of death in patients treated with the cyclophosphamide-methotrexate-fluorouracil chemotherapeutic regimen [67]. High SDC1 expression was reported in triple-negative invasive ductal breast carcinomas compared to normal breast tissue [68]. SDC1 expression was also strongly correlated with patient overall survival [68]. Evaluation of SDC1 expression in invasive ductal carcinoma indicated that cytoplasmic expression of SDC1 was positively correlated with WNT1 (a proto-oncogene) and membranous expression of SDC1 was positively correlated with p16 (a tumor-suppressor protein) [69]. Another study found that SDC1 expression was significantly increased in invasive breast cancer cases, suggesting that it may serve as a useful prognostic biomarker for aggressive breast cancer [33]. A tissue microarray of invasive ductal breast carcinoma specimens indicated high expression of SDC1 in the breast epithelium of more than half of the patients, whereas stromal expression was observed in only one third of the patients. Moreover, a significant correlation was found between the loss of epithelial SDC1 expression in high-grade tumors. These findings suggest that lack of SDC1 epithelial expression is a strong prognostic marker in breast carcinomas [70]. Tiemann and colleagues studied the role of SDC1 in ductal carcinoma *in situ* of the breast (DCIS) [71]. Tumor grade was found to be related to the proportion of SDC1-positive cells, rather than to the intensity of SDC1 staining. In the same study, estrogen receptor (ER) expression did not affect the staining intensity of SDC1, but negatively correlated with the percentage of SDC1-positive cells. Further findings showed that expression of progesterone receptor (PR) was positively influenced by both the intensity of staining and the percentage of SDC1-positive cells. These results suggest a potential role of SDC1 in the pathogenesis of DCIS [71]. Additional studies compared expression of SDC1 in breast cancer cases with and without distant organ metastasis. SDC1 expression was significantly correlated with a higher histological grade. In addition, HER2 subtype and triple-negative carcinomas showed significantly higher SDC1 levels than those of control cases. Importantly, high SDC1 expression had a negative impact on both overall and disease-free survival rates [72].

In another study, SDC1 expression was detected in approximately 70% of breast cancer cases and found to correlate with tumor grade. The presence of SDC1 in high-grade tumors was associated with the absence of SDC4 [73]. Further evidence indicated strong staining of SDC1 in DCIS tumor samples, which was associated with E-cadherin and c-Met expression [46]. In addition, expression of SDC1 and SDC4 was correlated with the Ki-67 mitosis index, suggesting a role in breast cancer cell proliferation. In addition, SDC1 and SDC4 expression was correlated with negative ER status and aggressive phenotypes [74]. Strong SDC1 staining was observed in more than 80% of neoplastic cells and was associated with increased mortality risk. In addition, there was a strong negative correlation between SDC1 expression and extracellular matrix proteins, suggesting that SDC1 promotes tumor progression by interacting with extracellular matrix components and impacting breast cancer tissue remodeling [75]. In another study, the expression of SDC1 was equivalent in the epithelium and stroma of breast tumors, but epithelial SDC1 expression was associated with negative ER status while stromal SDC1 expression was associated with positive ER status [76]. Moreover, loss of epithelial or stromal SDC1 expression was associated with a more favorable 10-year overall survival rate [76]. These findings indicate that SDC1 is expressed at high levels in breast cancer and its expression is associated with aggressive phenotypes and poor clinical outcomes.

#### Cervical cancer

Most cervical cancer tissues assessed to date have been shown to be SDC1-positive, and localization of SDC1 in the cytoplasm was associated with better patient survival. In addition, the change of SDC1 expression in cervical cancers was not caused by copy number alteration of the gene [77]. The progression of cervical intraepithelial neoplasm to early invasive cancer was found to correlate with reduced levels of SDC1 [78]. In another study, biopsies obtained from patients treated for primary invasive cervical carcinoma showed that SDC1 expression is associated with histological differentiation grade and squamous histology, but the expression does not predict clinical outcome [79].

#### Colorectal cancer

Recent reports have indicated that colorectal patients have higher serum levels of soluble SDC1 compared to healthy adults, which correlates with poor survival [80]. Patients with high SDC1 serum levels were also less responsive to 5-fluorouracil, oxaliplatin, irinotecan, cisplatin, or paclitaxel chemotherapy treatments [80]. Further studies revealed that SDC1 is expressed at the basolateral borders of normal colonic epithelial cells; however, in adenocarcinoma cells, SDC1 was found to be present around epithelial cell membranes and in the cytoplasm [81]. In approximately 90% of adenocarcinomas examined, SDC1 expression was absent, and this correlated with lymph node metastasis. Stromal SDC1 was expressed in a small fraction of tumors. These findings emphasize that the loss of tumor SDC1 may be a potential prognostic biomarker for human colon adenocarcinomas [81]. In another study, the expression of epithelial SDC1 was observed in over 90% of colorectal cancer specimens and was associated with lower histological grade and a less advanced clinical stage [82]. Expression of stromal SDC1 was observed in 58% of specimens, but expression did not significantly correlate with clinical outcome [82]. Taken together, these studies indicate that SDC1 expression may be a useful biomarker for evaluating the stage and grade of colorectal tumors. The lack of consistency between studies may be related to patient selection or methodological differences, and therefore larger studies are needed to further evaluate the prognostic impact of SDC1 in patients with colorectal tumors.

#### Endometrial cancer

In endometrial cancer, epithelial SDC1 expression was significantly lower in advanced stage, high grade, and lymph node metastatic disease [83]. In contrast, stromal SDC1 expression was significantly higher in high-grade tumors [83]. Moreover, SDC1 expression was totally absent in poorly differentiated endometrial cancer tissues, while it was abundant in normal endometrial and highly differentiated malignant tissues [84].

#### Gallbladder cancer

Epithelial SDC1 was observed in approximately half of gallbladder cancer cases evaluated, and its expression was associated with lymph node metastasis. This study also found that patients with positive SDC1 expression had a significantly shorter survival time than patients with undetectable expression [85].

#### Gastric cancer

Loss of epithelial SDC1 expression as well as high stromal SDC1 expression was associated with unfavorable prognosis in gastric cancer [86]. Additional studies showed that loss of epithelial SDC1 expression was associated with high stromal SDC1 expression, higher tumor grade, poor overall survival, and nodal metastases [87]. Therefore, stromal and epithelial SDC1 expression might have some prognostic impact in gastric cancer. However, these findings are not consistent and require further investigation.

#### Glioma

Higher gene and protein levels of SDC1 were detected in glioma tissues compared to controls. Moreover, SDC1 expression was increased in high-grade tumors, and the overall survival rate of SDC1 positive patients was significantly lower than that of SDC1 negative patients [88].

#### Laryngeal cancer

SDC1 expression was detected in all laryngeal cancer specimens examined by Klatka and colleagues, and expression was significantly correlated with histological grade and patient survival rate [89]. Additional investigation indicated that tumors with intermediate or strong staining for SDC1 were associated with higher overall survival than tumors with no or low SDC1 expression [90].

#### Liver cancer

In patients with advanced hepatocellular carcinoma (HCC), serum levels of SDC1 were increased compared to those without HCC or with early HCC [91]. High serum SDC1 levels were significantly associated with greater risk of tumor recurrence and decreased overall survival in patients with early HCC and with advanced HCC, respectively [91]. Additional studies showed reduced expression of the SDC1 gene and protein in metastatic HCC patients compared to those with non-metastatic disease [92]. Thus, the loss of SDC1 expression could be a characteristic feature of HCC with high metastatic potential [92].

SDC1 protein and gene expression levels were also assessed in intrahepatic cholangiocarcinomas and normal bile duct epithelial cells [34]. Intrahepatic cholangiocarcinoma cells showed membranous and cytoplasmic expression of SDC1, while normal epithelial cells showed restricted basolateral membranous expression. In cancerous tissues, the distribution of SDC1 mRNA was similar to that of the protein, suggesting that SDC1 expression in intrahepatic cholangiocarcinoma is regulated at the transcriptional level. Moreover, loss of SDC1 expression in carcinoma was associated with poor differentiation and lymph node metastases [34].

#### Lung cancer

In lung cancer patients, high serum SDC1 and bFGF levels were associated with poor outcomes at the time of diagnosis [93]. In another study, evaluation of SDC1 expression in squamous cell lung carcinoma patients showed higher expression of SDC1 in well-differentiated cancers than in moderately or poorly differentiated tumors [94]. Cancers with high SDC1 expression were associated with more favorable overall survival, suggesting that loss of SDC1 expression occurs as a result of histological dedifferentiation and that low SDC1 expression is associated with unfavorable outcomes in squamous cell carcinoma of the lung [94].

#### Mesothelioma

Studies of the expression of SDC1 protein in mesothelioma tumors and cell lines revealed strong SDC1 expression in epithelial mesotheliomas and in epithelial components of biphasic mesotheliomas, while expression was reduced during sarcomatoid differentiation [95]. Moreover, SDC1 expression was associated with longer overall survival in patients with mesotheliomas compared to patients with no or low SDC1 expression [95]. In another study, SDC1 was detected in pleural effusions, but not in sera of patients with pleural metastatic disease and malignant mesothelioma [67]. These findings distinguish malignant and benign diseases and suggest that SDC1 expression levels may be a prognostic factor that can predict differences in survival [67].

#### Nasopharyngeal carcinoma and oral cancer

An analysis of SDC1 and c-Met in samples from nasopharyngeal carcinoma patients by immunohistochemical staining indicated that high coexpression of c-Met and SDC1 was adversely correlated with patient survival [96]. Normal oral mucosa has been shown to express moderate-to-high levels of SDC1, which is reduced or abolished in carcinomas [97]. In another study, SDC1 was found to be mainly expressed in the stromal cells, and this pattern was associated with poor prognosis of ameloblastomas, keratocystic odontogenic tumors [98]. SDC1 expression was significantly higher in normal controls than in specimens from patients with mild, moderate, or severe dysplasia as well as invasive squamous cell carcinoma; however, no significant difference was found between different tumor grades [99]. In another study, approximately 90% of oral squamous cell carcinoma cases showed negative or weak SDC1 staining. Patients with intermediate or strong staining intensity for SDC1 had a significantly better prognosis than patients with negative or weak staining intensity [100]. SDC1 expression was decreased in more than 80% of oral carcinoma cases examined, but positive stromal SDC1 staining proved to be a significant risk factor of recurrence and tumor-specific death within a 24-month period after surgery, suggesting stromal expression of SDC1 is a reliable indicator of an adverse prognosis in oral carcinomas [101]. In another study, SDC1 levels were increased in response to cytostatic treatment, which proved to be an important predictive factor and a clear forecast of a good prognosis [102]. Taken together, these results suggest that reduced cellular SDC1 or increased stromal SDC1 expression can be useful prognostic factors in oral cancers. However, a full understanding of the contrasting characteristics between epithelial and stromal components requires further studies.

### Ovarian cancer

Patients with advanced ovarian cancer exhibit significantly lower epithelial SDC1 expression and significantly higher stromal SDC1 expression in reciprocal pattern compared to normal controls [103]. Additional studies have evaluated the expression of syndecans in benign and malignant ovarian tumors and found that SDC2, -3, and -4 are expressed in normal, benign, and malignant ovarian tissues [104]. In contrast, SDC1 was absent in normal ovarian tissues, but present in epithelial and stromal cells of benign and borderline tumors. In addition, the expression of stromal SDC1 was a poor prognostic factor of overall survival in patients with ovarian cancer [104].

#### Pancreatic cancer

Epithelial SDC1 has been observed in most human pancreatic carcinoma samples evaluated to date, and expression is predictive of a more favorable prognosis in patients undergoing curative surgery. In addition, stromal SDC1 expression was weak or negative in over 60% of the tumors evaluated, and lack of stromal expression predicted a better prognosis in these patients [105].

#### Prostate cancer

Early studies have shown that syndecans are expressed in the epithelial cells of prostate cancer patients [106]. SDC1 showed basolateral membrane localization, whereas SDC2 was preferentially expressed in basal cells. Another study found that the expression patterns of SDC1 and SDC2 changed to a granular-cytoplasmic localization in prostate cancer samples [106]. Moreover, SDC1 was detected by immunostaining of a tissue microarray in approximately one third of patients with localized prostatic adenocarcinoma who had been treated with radical prostatectomy and bilateral lymphadenectomy [107]. SDC1 expression was also associated with lymph node metastases and aggressive progression after surgery. Further studies showed altered expression of SDC1 protein in specimens obtained from normal, benign, and malignant prostate tissues [108]. SDC1 overexpression in human prostate cancer was also predictive of early recurrence and was associated with tumor-specific survival, high Gleason grade, the Ki-67 mitosis marker, and Bcl-2 overexpression [109]. Together, these findings suggest that expression of SDC1 can be used as a prognostic marker for patients with localized and advanced prostate cancer.

#### Squamous cell carcinoma of the head and neck and thyroid cancer

Analysis of primary squamous cell carcinoma of the head and neck in patients treated with surgery and post-operative radiotherapy has shown low SDC1 expression [110], which was associated with low grade of differentiation, large tumor size, increased nodal metastases, high clinical stage, and unfavorable overall survival [110]. SDC1 expression in these tumors was also associated with higher overall and recurrence-free survival compared to no or low SDC1 expression [111]. A tissue microarray analysis of SDC1 expression in papillary carcinomas of the thyroid indicated that SDC1 was mainly expressed in the cytoplasm of epithelial cells and stroma of papillary carcinomas of the thyroid [113].

### SDC1 as a prognostic biomarker in hematological tumors

#### Chronic lymphocytic leukemia

Studies assessing the correlation between soluble SDC1 in plasma and clinical outcome in patients with chronic lymphocytic leukemia have shown that soluble SDC1 levels were significantly higher in these patients compared to healthy control subjects. In addition, high levels of soluble SDC1 were also associated with shorter overall survival [112].

#### Diffuse large B-Cell lymphoma

Multiple studies have detected SDC1 in diffuse large B-cell lymphoma [113, 114]. Tumor biopsies of diffuse large B-cell lymphoma patients were examined for SDC1 expression and results tested positive in 30% of poor overall survival [113, 114], indicating aberrant SDC1 expression correlates with poor clinical outcome.

#### Multiple myeloma

Several studies have shown higher levels of soluble SDC1 in multiple myeloma patients compared to healthy controls [115–117]. Baseline levels of soluble SDC1 at the time of diagnosis in patients who responded to chemotherapy were lower than non-responders; however, baseline levels of SDC1 did not predict therapeutic response in those patients [115]. High levels of soluble SDC1 and lower expression of cellular SDC1 at the time of diagnosis are negative prognostic factors for multiple myeloma [116]. In a cohort of Korean patients diagnosed with multiple myeloma, soluble SDC1 levels correlated with disease stage and characteristics [118]. In addition, high soluble SDC1 levels detected in Korean subjects were associated with poor survival [118]. Further studies showed that soluble SDC1 levels were elevated in the sera of multiple myeloma patients treated with high-dose chemotherapy and subsequent autologous transplantation [119]. In another study, the extent to which soluble SDC1 levels fell from presentation to the plateau phase represented a prognostic predictor in multiple myeloma patients [56]. In a comparative study of blood dyscrasias, multiple myeloma patients showed higher serum SDC1 levels than patients with plasmocytoma or monoclonal gammopathy [120]. In addition, serum SDC1 levels were diminished in patients who responded well to chemotherapy, whereas no change was observed in non-responders [120]. When SDC1 expression was analyzed in normal bone marrow or bone marrow from multiple myeloma and B-cell lymphoma patients, SDC1 was found to be expressed predominantly in normal and neoplastic plasma cells. Moreover, high SDC1 expression was detected in all multiple myeloma cases examined, whereas all B-cell lymphomas were completely negative.

Evaluation of SDC1 levels in the bone marrow of multiple myeloma patients showed much higher levels than circulating SDC1 levels in peripheral blood [121]. Nevertheless, SDC1 blood and bone marrow levels were positively correlated with microvessel density, HGF levels, and reduced survival [121]. Serum SDC1 and bFGF/FGF2 levels were elevated in multiple myeloma patients before treatment compared to the control group [122]. Baseline assessment of SDC1 and bFGF/FGF2 serum levels showed higher levels of both markers, which was associated with shorter survival than patients with normal levels [122]. In the same study, myeloma patients responding to chemotherapy treatment showed reduced SDC1 levels [122]. Bone marrow levels of soluble SDC1 and HGF were elevated in multiple myeloma patients compared to control subjects [117]. In addition, HGF existed in a complex form with soluble SDC1 in pleural effusions, suggesting an important role of soluble SDC1 as a carrier for HGF in the pathology of myeloma [117]. Taken together, various studies have demonstrated SDC1 as a potential biomarker for multiple myeloma. Findings from these studies indicate that soluble SDC1 levels may be a prognostic tool in multiple myeloma patients for diagnosis, prognosis, and treatment response.

### Putative roles of SDC1 in Hodgkin's lymphoma

Hodgkin's Lymphoma (HL) is characterized by the presence of cancerous Hodgkin-Reed-Sternberg (HRS) cells embedded in a background of immune, inflammatory and stromal cells [123]. These cells secrete a plethora of cytokines and growth factors in the tumor microenvironment that lead to tumor growth and dissemination. SDC1 acts to potentiate the signaling of cancerous and stromal cells in the tumor microenvironment. Serum levels of SDC1 were higher in HL specimens compared to a control group [124]. In another study, B-cell markers, including SDC1, were expressed in 38% of classical HL cases [125]. The following sections highlight three potential pathways involving SDC1 in HL pathogenesis.

#### SDC1 and HGF

**Figure 2:**
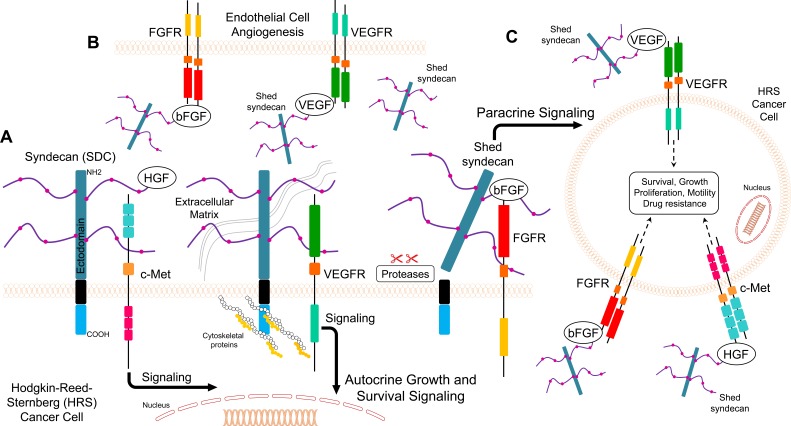
Model for putative roles of SDC1 in Hodgkin's lymphoma **A.** SDC1 facilitates autocrine interaction between growth factors and their cognate receptors and enhances mitogenic signaling in Hodgkin-Reed-Sternberg (HRS) cancer cells **B.** Shed SDC1 (sSDC1) binds to growth factor VEGF and bFGF complexes with VEGFR and FGFRs in endothelial cells and promotes angiogenesis **C.** Shed SDC1 (sSDC1) binds to growth factors to interact with cognate receptors on another HRS cell (paracrine effect).

It has been reported that HL patients have increased serum levels of HGF, which correlates with advanced stages of the disease [126]. SDC1 binds to HGF, which potentiates c-Met downstream signaling by activating the PI3K and ERK pathways (Figure 2) [127]. Moreover, it has been reported that c-Met is expressed by subsets of Hodgkin Reed Sternberg (HRS) cells, and HGF is secreted in the tumor milieu, suggesting an autocrine effect in HL pathogenesis [128]. In another study, changes in plasma heparanase levels correlated with the response to treatment in pediatric patients diagnosed with HL [57]. Heparanase induced HGF expression and shedding of SDC1 through the upregulation of matrix metaloprotease-9 (MMP-9) and urokinase-type plasminogen activator (uPA) [129]. These findings suggest that SDC1 or soluble SDC1 binds to HGF to facilitate binding and activation of its receptor (Figure 2).

#### SDC1 and VEGF

Angiogenesis is a crucial process during the progression of hematological malignancies, including HL [126]. High serum levels of VEGF were detected in the sera of HL patients [126]. Moreover, the levels of VEGF and VEGF receptor in HL patients were significantly higher than the levels in non-Hodgkin's lymphoma (NHL) patients [130]. In another study, the overexpression of VEGF was approximately 70% of cases of classical HL and 30% of nodular lymphocyte predominance HL, and all neoplastic HRS cells [131]. In a separate study, VEGF-A, VEGF receptor-1, and VEGF receptor-2 were expressed in HRS cells from patients with classical HL [132]. When heparanase expression was high in the tumor, soluble SDC1 formed a complex with VEGF, which activated VEGF receptors on adjacent endothelial cells (Figure 2) [30]. These findings suggest that SDC1 or soluble SDC1 enhance VEGF binding to the VEGF receptor, thus promoting angiogenesis (Figure 2).

#### SDC1 and bFGF/FGF2

Unlike other growth factors, FGFs act with HSPGs (such as SDC1) to activate FGF receptors and induce downstream signaling responses [133, 134]. As described above, the binding of bFGF/FGF2 and an HSPG to the extracellular domain of the FGF receptor induces receptor autophosphorylation. This process leads to the phosphorylation of docking molecules, such as Shc, phospholipase-Cγ, STAT1, Gab1, and FRS2α, which are regulators of the Ras/MAPK and PI-3K/Akt signaling pathways (Figure 2) [133]. It has been reported that serum levels of bFGF were elevated and correlated with the stage of different hematological malignancies [135]. In addition, the event-free survival rate was higher in NHL patients who had lower bFGF levels [136]. Another study showed that high serum levels of bFGF are associated with a poor outcome in NHL patients [137]. It has been reported that HRS cells and stromal cells secrete bFGF, which stimulates fibrosis in the nodular sclerosis (NS) subtype of HL [123]. In addition, serum bFGF levels were significantly higher in HL patients than in healthy individuals and correlated with the clinical outcome of HL [126]. Expression levels of FGFs and their receptors were high in HL patient samples, while their expression in HL cell line cultures was stimulated in response to paracrine factors [133]. The expression of bFGF and SDC1 in HL suggests that they play a role in maintaining the growth of HL cells [138]. The association of high serum levels of both SDC1 and bFGF with poor outcome in lung cancer has been reported [93]. Furthermore, Kyrtsonis and colleagues demonstrated that patients who had high serum levels of both SDC1 and bFGF had a shorter overall survival than patients with normal levels, and responders to treatment regimens showed reduced SDC1 levels [122]. In addition, a bioinformatics analysis showed overexpression of bFGF and SDC1 in HL cell lines that were originally derived from primary HRS cells isolated from extranodal sites of refractory or relapsing HL patients [138]. The expression levels of bFGF and SDC1 protein were significantly elevated in HL patient samples compared to NHL sections and normal lymph node controls [138]. Furthermore, all HL tissue samples overexpressed *FGF2* and *SDC1* genes, and the group with a poor outcome had a 24-fold higher expression of *FGF2* and 56-fold higher expression of *SDC1* than the group with a favorable outcome. Strong immunostaining of bFGF and SDC1 was also reported in the poor outcome HL group [138]. Taken together, these findings suggest that simultaneous high levels of bFGF and SDC1 correlate with a poor prognosis in HL patients.

### Syndecan as a therapeutic target in clinical settings

**Figure 3:**
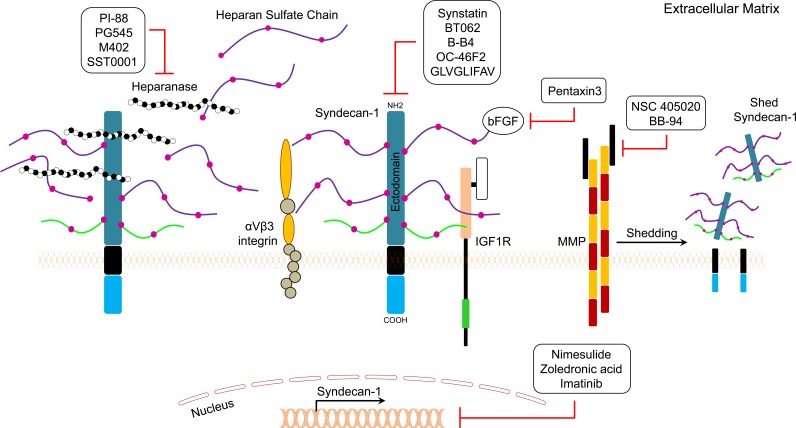
General mechanisms of action of SDC1 pathway inhibitors are depicted

Based on the numerous roles in cancer pathology, SDC1 is an attractive molecular target for therapeutic strategies. Quantification of SDC1 is necessary in basic discovery research as well as in clinical practice. *In vitro* diagnostics and technologies that allow for the specific detection and precise quantification of SDC1 continue to evolve. Today, selected clones that produce monoclonal antibodies can be cultured to produce SDC1-specific antibodies. This part of the review sheds light on recent advances in *in vitro* diagnostics as well as research-use only diagnostics (Table 4). It also summarizes SDC-targeting therapeutic modalities (Figure 3; Table 5) and the progress in clinical trials related to the SDC pathway (Table 6).

**Table 4:** *In vitro* diagnostics (IVD) and research use only (RUO) detection methods for SDC1

Diagnostic Type	Source (Host)	Reactivity	Manufacturer	Market Status	Applications	Cancer Type	Ref
Antibody (Clone B-A38)	Mouse	Amino acid sequence (18-218) of human SDC1	Zeta Corporation (CA, USA) Cell Marque (CA, USA) Biocare Medical (CA, USA)	Class I IVD	IHC Flow cytometry	Plasma cell malignancies	[170]
Antibody (Clone MI15)	Mouse	Ectodomain of human SDC1	Dako (CA, USA) Genemed Biotechnologies (CA, USA)	Class I IVD	IHC Flow cytometry	Plasma cell malignancies	[170, 171]
Antibody (Clone ZMD.289)	Rabbit	Middle region of human SDC1 ectodomain	Thermo Fisher Scientific (MA, USA)	Class I IVD	IHC	Plasma cell malignancies	[172]
Antibody (Clone EP201)	Rabbit	Ectodomain of human SDC1	Diagnostic Biosystems (CA, USA)	Class I IVD	IHC	Plasma cell malignancies	*
Antibody (Clone B-B4)	Mouse	Amino acid sequence (90-93) of human SDC1	Miltenyi Biotech (Germany)	RUO	IHC Flow cytometry	N/A	[170, 173]
Antibody (Clone DL-101)	Mouse	Ectodomain of human SDC1	Biolegend (CA, USA) Affymetrix eBioscience (CA, USA) Thermo Fisher Scientific (MA, USA)	RUO	IHC Flow cytometry	N/A	[170]
Antibody (Clone CLB-1D4)	Mouse	Ectodomain of human SDC1	Life Technologies (CT, USA) Lifespan Biosciences (WA, USA)	RUO	IHC Flow cytometry	N/A	[170, 174]
Antibody (Clone SP152)	Rabbit	Recombinant human SDC1	Pierce Antibodies (MA, USA) Abcam (UK) Lifespan Biosciences (WA, USA)	RUO	IHC	N/A	*
Sandwich ELISA	Mouse	Natural and recombinant soluble human SDC1	Abnova (Taiwan) BioVendor R&D (Czech Republic)	RUO	ELISA	N/A	[58]

IVD: *in vitro* diagnostic; RUO: research use only;

* information taken by contact with company.

**Table 5:** Agents targeting SDC1 in cancer

**Target**	**Agent**	**Manufacturer**	**Agent Type**	**Characteristics**	**Status (Trial #)**	**Indication**	**Ref**
**Extracellular domain of SDC**	Synstatin	-	Peptide	Competes with SDC1 for binding to EGFR/α6β4 integrin complex	-	Breast cancer, multiple myeloma	[140]
	BT-062-DM4 (Indatuximab Ravtansine)	Biotest Pharmaceuticals Corporation (FL, USA)	Antibody conjugated to a cytotoxic agent (DM4)	Selectively causes SDC1+ cancer cell death	Phase I/II (NCT01638936) All patients achieved stable disease	Breast cancer, bladder cancer, multiple myeloma	[142]
	B-B4 conjugated to I^131^	Centre René Gauducheau (France)	Antibody conjugated to I^131^	Selectively kills SDC1+ cancer cell	Phase I (NCT01296204) ¼ of patients exhibited partial response	Multiple myeloma	[143]
	OC-46F2	-	Antibody	Reduces SDC1/VEGFR-2 interaction	-	Melanoma, ovarian cancer	[144]
	GLVGLIFAV	-	Cytotoxic T lymphocytes	Selectively target SDC1+ cells	-	Multiple myeloma	[145]
**Shed SDC**	NSC 405020	-	Protein	Inhibits MT1-MMP homodimerization	-	Breast cancer	[146]
	BB-94	-	Small molecule	MMP inhibitor	Phase III (discontinued)	Breast cancer	[147]
	PI-88	Progen Pharmaceuticals (Australia)	Small molecule	Heparanase inhibitor	Phase III (NCT01402908) (ongoing)	Liver cancer	[150]
	PG545		Small molecule	Heparanase Inhibitor	Phase I NCT02042781 (ongoing)	Solid tumors	[175]
	M402		Small molecule	Heparanase Inhibitor	Phase I/II NTC01621243 (ongoing)	Pancreatic cancer	[176]
	SST00001	-	Modified heparin	Heparanase Inhibitor	Phase I (NCT01764880) (ongoing)	Multiple myeloma	[177]
	Pentraxin-3	-	Protein	FGF2 antagonist	-	Pancreatic cancer	[157]
**Genetic expression of SDC**	All-trans retinoic acid	-	Micronutrient	Inhibits BαP-induced shedding	-	Lung cancer	[155]
	Nimesulide	-	Small molecule	Downregulates SDC1, SDC2 expression	-	Primary effusion lymphoma	[159]
	Zoledronic acid (Zometa)	Novartis (Switzerland)	Small molecule	Downregulates SDC1, SDC2 expression	-	Breast cancer	[161]
	Imatinib (Glivec)		Small molecule	Downregulates SDC2, SDC4 expression	-	Breast cancer	[162]

SDC: Syndecan; EGFR: epidermal growth factor receptor; VEGFR: vascular endothelial growth factor receptor; MT1-MMP: membrane type 1 metalloprotease; FGF2: fibroblast growth factor-2.

**Table 6:** Clinical trials related to the SDC1 pathway

Clinical Trial Description (Trial #)	Participants #	Start Date/ Trial Status	Originator	Sponsor	Agent Type / Mechanism of Action	Study Type/ Purpose	Ref
Phase I/II multi-dose escalation study of BT062 (Indatixumab Ravtansine) in combination with lenalidomide and dexamethasone in subjects with relapsed multiple myeloma (NCT01638936)	49	July 2012/ recruiting - ongoing	Biotest Pharmaceutical Co. (FL, USA)		Monoclonal SDC1 antibody coupled with cytotoxic agent (DM4)/ inhibits tubulin polymerization	Interventional, optimal dosage determination, evaluation of response, dose escalation study	[142]
Phase I open-label, dose-finding study evaluating safety and pharmacokinetics of FPA144 in patients with advanced solid tumors (NCT02318329)	100	November 2014/ recruiting - ongoing	Five Prime Therapeutics, Inc. (CA, USA)		Monoclonal FGFR2 antibody/ FGFR2 antagonist	Interventional, safety of escalating doses	[178]
Phase III, pivotal study of SGN-35 (Adcetris®; Brentiximab Vedotin) in treatment of patients with relapsed or refractory Hodgkin lymphoma (NCT00848926)	102	February 2009/ active, not recruiting - ongoing	Seattle Genetics (WA, USA)		Immunoconjugate monoclonal antibody against integrin αvβ3 / tubulin polymerization inhibitor	Interventional, best clinical outcome, 75% of participants responded to treatment	[179]
Phase I study assessing safety and tolerability of SST0001 (Roneparstat) in advanced multiple myeloma (NCT01764880)	30	November 2012/ recruiting - ongoing	Sigma Tau Research Switzerland SA (Italy)		Chemically-modified heparin/ heparanase inhibitor	Interventional, maximum tolerated dose with primary purpose of treatment	*
Phase I study evaluating the toxicity, pharmacokinetics and biological effect of intravenous Bevacizumab (Avastin™) in combination with escalating doses of oral AZD2171 (Recentin™; Cediranib) for patients with advanced malignancies (NCT00458731)	57	May 2007/ completed	Bevacizumab: Genentech (CA, USA); Recentin: AstraZeneca (DE, USA)	National Cancer Institute (MD, USA)	Bevacizumab: VEFG-A monoclonal antibody/ VEGFR signaling inhibitor Recentin: quinazoline small molecule / VEGFR antagonist	Interventional, safety study with primary purpose of treatment	[180]
Phase I dose escalation study of OMP-54F28 (Ipafricept) in subjects with solid tumors (NCT01608867)	36	June 2012/ active, not recruiting - ongoing	OncoMed Pharmaceuticals, Inc. (CA USA)	Bayer Healthcare Pharma. (Germany)	Immunoglobulin fusion proteins/ Wnt pathway inhibitor, targets R-spondin	----------	[181]
Phase II study of VS-6063 (Defactnib) in patients with KRAS mutant non-small cell lung cancer (NCT01951690)	150	September 2013/ recruiting - ongoing	Pfizer (New York, NY USA)// Verstern, Inc. (MA USA)		Small molecule/ FAK Inhibitor	Interventional, PFS improvement was measured every 12 weeks	[182]
Phase I open-label dose escalation study of GSK2256098 in subjects with solid tumors (NCT01138033)	138	July 2010/ recruiting - ongoing	GlaxoSmithKline (NJ, USA)		Small molecule/ FAK Inhibitor	Interventional, safety and tolerability with primary purpose of treatment	[183]
Phase I/II study to evaluate the safety and efficacy of M402 (Necuparanib) in combination with nab-paclitaxel and gemcitabine in patients with metastatic pancreatic cancer (NCT01621243)	180	May 2012/ recruiting - ongoing	Momenta Pharmaceuticals (MA, USA)		Necuparanib: glysoaminoglycan / heparanase Inhibitor	Interventional, safety and overall survival measured in response to treatment	[153]
Phase I/II Study of PI-88 (Muparfostat) in malignancies (Phase I), and in Advanced Melanoma (Phase II) (NCT00068172)	88	March 2009/ completed	Progen Pharmaceuticals (Australia)		Mannan, oligosaccharide / FGF inhibitor; heparanase inhibitor; VEGF inhibitor	Interventional, progression-free survival	[151]
Phase I, open-label study of the safety and tolerability of PG545 in patients with advanced solid tumors (NCT01252095)	4	January 2011/ Terminated	Progen Pharmaceuticals (Australia)		Chemically modified heparin sulfate/ heparanse inhibitor	Interventional, maximum tolerated dose was measured	[152]
Phase I study of intrapleural batimastat (BB-94) in the treatment of malignant pleural effusions	18	----------	British Biotech (UK)		Amino acids/ matrix metalloprotease inhibitor	Interventional; optimal dosage determination	[149]
Study of Chimeric CD138 antigen receptor-modified T cells in relapsed and/or refractory multiple myeloma patients (NCT01886976)	10	June 2013 – ongoing / recruiting	Chinese PLA General Hospital		Chimeric anti-CD138 antigen receptor-modified T cells	Interventional, safety and tolerability with primary purpose of treatment	[163]

* https://clinicaltrials.gov/ct2/show/NCT01764880

Synstatin is a short peptide that mimics the sequence of the SDC1 extracellular domain [139, 140]. This peptide antagonizes the SDC1 extracellular domain, which is responsible for capturing and activating αvβ3 or αvβ5 integrins and the insulin-like growth factor-I (IGF-I) receptor (Figure 3). Synstatin competitively displaces the integrin and IGF-I receptor kinase from SDC1 and inactivates the complex, which makes it a promising anti-angiogenic agent [139, 140]. BT062 (Indatuximab Ravtansine) is an antibody-drug conjugate that is comprised of the anti-SDC1 chimerized monoclonal antibody and the cytotoxic agent DM4. Once bound to SDC1 on the cell, the conjugate is internalized and releases DM4, which consequently leads to cell death. A study was conducted to evaluate the effect of BT062 on multiple myeloma patients heavily pretreated with revelimid, thalidomide, velcade, or carlfilzomid [141, 142]. BT062 was well-tolerated in patients and 4% achieved partial response, 8% had a minor response, while 38% showed stable disease [141]. B-B4 is a monoclonal IgG1 antibody conjugated to cytotoxic drugs or radioactive isotopes. A phase I/II radioimmunotherapy study using B-B4 conjugated to iodine-131 was conducted in refractory multiple myeloma patients [143] and significantly improved clinical outcome than the control group, suggesting that targeted radioimmunotherapy is feasible using an anti-SDC1 monoclonal antibody [143].

OC-46F2 is a fully human recombinant that specifically recognizes the SDC1 ectodomain (Figure 3) [144]. OC-46F2 was found to inhibit SDC1 distribution in the tumor milieu, thus preventing vascular maturation and tumor growth in experimental human melanoma and ovarian carcinoma models [144]. GLVGLIFAV is an SDC1-specific peptide that is recognized by cytotoxic T lymphocytes generated *ex vivo* using an HLA-A2-specific SDC1 epitope against multiple myeloma cells [145]. The GLVGLIFAV peptide induces antigen-specific cytotoxic T lymphocytes, which might be useful for the treatment of multiple myeloma patients with peptide-based vaccines or cellular immunotherapy strategies [145]. Membrane type 1 metalloprotease (MT1-MMP) is a transmembrane metalloprotease that stimulates the shedding of several proteoglycans, such as SDC1. NSC 405020 is a small molecule inhibitor that inhibits MT1-MMP homodimerization, thus blocking its pro-tumorigenic activity *in vivo* [146]. BB-94 (Batimastat) is a potent, broad spectrum small molecule inhibitor of MMP [147]. Treatment of cells with BB-94 suppressed SDC1 shedding and induced accumulation of SDC1 on the cell surface [148]. On patients with cytologically positive malignant pleural effusions in Phase I study of intrapleural BB-94, BB-94 peaked after 4 h, and remained in plasma for up to 12 weeks, indicating that the intrapleural BB-94 was well tolerated, with evidence of local efficacy [149].

PI-88 is a polysaccharide compound with anti-heparanase activity (Figure 3) [52]. In a phase II study in hepatocellular carcinoma (HCC) patients, PI-88 treatment was administered over nine, 4-week treatment cycles, followed by a 12-week treatment-free period. PI-88 at 160 mg/day was tolerable and effective as an adjunct therapy for post-surgery HCC [150]. In another phase I study in patients with advanced solid tumors, the compound was found to be well-tolerated when administered for 4 consecutive days bimonthly or weekly at the recommended dose of 250 mg/day [151]. One melanoma patient had a partial response, and nine patients maintained a stable disease state for more than six months [151]. M402 is another modified heparin compound similar to SST0001, a chemically modified heparin to inhibit myeloma growth [152]. PG545 is a fully sulfated, synthetic tetrasaccharide that exerts anti-heparanase activity and has shown promising results against many cancer cells[152]. In ovarian cancer cells, PG545 showed synergistic inhibition of growth and migration in combination with paclitaxel and cisplatin [152]. M402 is smaller than SST0001 and has broader activity in binding to growth factors. M402 was found to be an effective anticancer agent in different cancer models [152]. M402 inhibits stromal activation and reduces tumor size in nude mice with human pancreatic cancer cells [153]. An ongoing Phase I/II study evaluating the safety and tolerability of M402 is currently being conducted in patients with metastatic pancreatic cancer [153]. SST0001 is a modified heparin with anti-heparanase activity that inhibits cancer cell growth and metastasis [154]. Results of *in vivo* studies showed that SST0001 effectively inhibited myeloma growth and diminished heparanase-induced shedding of SDC1 [154].

All-trans retinoic acid, an active metabolite of retinal, has been shown to exert anticancer activity against different cancer cells. It has been reported that benzo(α)pyrene induces accumulation of shed SDC1 in lung cancer [155]. One study examined the level of SDC1 expression and the chemopreventive effect of all-trans retinoic acid in a benzo(α)pyrene-induced lung cancer model in BALB/c mice. The results indicated that all-trans retinoic acid inhibited lung tumor development and reduced SDC1 expression in cancer cells [155]. It has been reported that bFGF/FGF2 induces shedding of SDC1 in cancer cells [156]. Pentraxin-3 is a bFGF antagonist that binds to bFGF with high affinity and prevents the binding of bFGF to its receptor [157]. Therefore, pentraxin-3 may be of value to inhibit SDC1 shedding induced by bFGF (Figure 3) [156].

Nimesulide is a cyclooxygenase-2 selective, non-steroidal anti-inflammatory drug [158]. Paul and colleagues reported that nimesulide treatment caused cell cycle arrest in primary effusion lymphoma cell lines, and this effect was accompanied by downregulation of SDC1 [159]. Zoledronic acid (Zometa^®^) is a third-generation bisphosphonate that inhibits SDC1 expression in cancer cells in a dose-dependent manner [160]. Moreover, zoledronic acid effectively inhibited growth, migration, and adhesion of human breast cancer cells, which was accompanied by downregulation of SDC1 and -2 [161]. Moreover, imatinib (Gleevec^®^) is a tyrosine kinase inhibitor of PDGF receptor, c-Kit, and Bcr-Abl. It exerts a significant inhibitory effect on the expression of SDC2 and SDC4 in cancer cells, which leads to suppression of cell growth ability, migration, and invasion (Figure 3) [162]. More recently, a clinical trial was conducted to evaluate the safety and efficacy of autologous T cells expressing an anti-CD138 chimeric antigen receptor (CART138) in patients with relapsed or refractory multiple myeloma [163] and results showed that CART-138 immunotherapy was well-tolerated with significant clinical benefits in multiple myeloma patients [163].

## CONCLUSIONS

SDC1 is a cell surface adhesion molecule that is essential for maintaining cell morphology and interactions with the microenvironment. SDC1 exerts specific functional roles by acting as a coreceptor, thus potentiating binding between growth factors and their membrane receptors. Proteolytic activity releases the extracellular ectodomain of SDC1, which harbors both the HS and CS chains, thus resulting in soluble/shed SDC1. Soluble SDC1 facilitates binding between growth factors and their receptors, or functions as a decoy receptor in other circumstances. SDC1 can be found in the cytoplasmic compartment as well as the nuclear compartment of cells. Nuclear SDC1 can activate gene transcription and result in distinct physiologic activities. In cancer, growing evidence indicates that deregulation of SDC1 contributes to the development and progression of different tumor types. The value of SDC1 as a prognostic marker for specific cancer types has been extensively evaluated in solid and hematological cancers. In addition, multiple reports have examined the prognostic impact of the cellular localization of SDC1. However, based on the data to date, it is difficult to directly correlate the levels of SDC1 expression with tumor characteristics and prognostic significance for all cancers in general and formulate personalized clinical treatment approaches. However, the concept of precision medicine can be implicated for specific cancer types, since higher or lower SDC1 expression is directly associated with more aggressive tumors and decreased patient survival in some cases. Such profiling may be useful for patient selection at the time of diagnosis or perhaps for relapsing patients. In addition, SDC1 expression is associated with a weaker response to chemotherapy for numerous solid tumors, including breast, colorectal, and prostate cancers. Therefore, appropriately targeting SDC1 in selected cancers may guide precision therapeutic options. The reciprocal expression signature of SDC1 whereby expression is reduced in tumor epithelium and increased in tumor stroma has been evaluated in multiple studies, and recent reports suggest that SDC1 plays a functional role in cancer-activated stromal components as well as in tumor progression in selected cancer types. Interestingly, most studies have shown distinct cellular expression patterns for SDC1, in which membranous and cytoplasmic expression profiles were different in tumor samples compared to control samples from different types of cancers. Therefore, it can be concluded that total expression levels as well as the cellular distribution of SDC1 should be evaluated together for the most informative prognostic tools. Evaluation of urinary levels of SDC1 in urinary bladder tumors may also be considered during the assessment of tumor severity. The value of circulating levels of SDC1 was not consistently associated with tumor grade or characteristics, but the combination of SDC1 and bFGF/FGF2 in patient serum has a strong association with tumor progression and prognosis in selected cancer types. In multiple myeloma, soluble SDC1 levels were found to be directly associated with the progression of disease, and therefore this association should be evaluated in other cancers as well. Multiple clinical trials are currently evaluating the safety and efficacy of pharmacologically targeting SDC1 in different types of cancer. Collectively, SDC1 represents an attractive molecular target for further evaluation in personalized cancer treatment. The identification and clinical validation of SDC1 as a new diagnostic and predictive biomarker will enable individualized therapeutic management for poor outcome cancer patients who are refractory to therapy or under high risk of relapse.
